# Cocreation to Facilitate Communication and Collaboration Between Multidisciplinary Stakeholders in eHealth Research and Development: Case Study of the CARRIER (Coronary Artery Disease: Risk Estimations and Interventions for Prevention and Early Detection) Consortium

**DOI:** 10.2196/45006

**Published:** 2023-10-24

**Authors:** Elizabeth Latuapon, Laura Hochstenbach, Dominik Mahr, Bart Scheenstra, Bas Kietselaer, Marieke Spreeuwenberg

**Affiliations:** 1 Department of Health Services Research Care and Public Health Research Institute Maastricht University Maastricht Netherlands; 2 Department of Marketing & Supply Chain Management School of Business and Economics Maastricht University Maastricht Netherlands; 3 Department of Cardiothoracic Surgery Cardiovascular Research Institute Maastricht Maastricht University Medical Centre Maastricht Netherlands; 4 Department of Cardiovascular Disease Mayo Clinic Rochester, MN United States; 5 Department of Cardiology Zuyderland Medical Centre Heerlen Netherlands

**Keywords:** eHealth, cocreation, stakeholder involvement, multidisciplinary collaboration, multidisciplinary, team dynamic, group dynamic, collaborate, collaboration, cardiovascular, personalized, personalization, cardiology, organizational, co-design, atherosclerosis

## Abstract

**Background:**

Collaboration with diverse stakeholders in eHealth research is fundamental yet complex. Stakeholders from various disciplines do not “speak the same language” and have different levels of power and interest, resulting in contrasting objectives, priorities, and expectations. An approach to constructive communication and collaboration is necessary to overcome this complex dynamic. Cocreation, known in the field of eHealth most often to involve end users, may also be suitable for facilitating stakeholder engagement and alignment.

**Objective:**

This paper provides insights into the application of cocreation, specifically in the early phases of research that focus on involving and aligning relevant stakeholders from different academic and professional backgrounds.

**Methods:**

The case for this study was a group discussion with members of a multidisciplinary consortium that works on developing a personalized eHealth intervention for atherosclerotic cardiovascular disease. Using stakeholder mapping, health and medicine experts, big data scientists, software developers, and an innovation manager (N=8) were invited to participate. The discussion was based on a user scenario and structured according to the Six Thinking Hats of de Bono, representing 6 different types of thinking. The discussion was recorded, transcribed verbatim, and analyzed thematically with the use of ATLAS.ti software.

**Results:**

First, informative and intuitive thinking served the preparatory purpose of familiarization with the project details and other participants. Second, positive and critical thinking constituted the body of the discussion and resulted in an in-depth conversation. Third, creative and organizational thinking were action oriented and focused on solutions and planning to safeguard future progress. The participants repeatedly reflected on various intervention-related themes, ranging from intervention content to technical functionalities and from legal requirements to implementation in practice. Moreover, project-related matters were discussed, including stakeholder management and time and budget constraints.

**Conclusions:**

This paper demonstrates how cocreation can be of value for multidisciplinary stakeholder engagement and alignment. Based on stakeholder mapping (with whom to discuss), a dream user scenario (what to discuss), and the Six Thinking Hats of de Bono (how to discuss), the participants shared information, discussed differences, searched for solutions, and moved toward a collective approach regarding intervention development. The lessons learned may further improve the understanding of how cocreation can contribute to multidisciplinary collaboration.

## Introduction

In the current context of a high chronic disease burden and limited financial and human resources, attention has been directed toward innovative solutions, such as eHealth, a field that represents technological innovations that aim to improve health and well-being [1-3]. It is known for its promise for improving health care efficiency and effectiveness, facilitating just-in-time services, and empowering patients and health care providers (HCPs) regardless of their location while remaining cost-effective [4,5]. eHealth is a rapidly growing field with innovations ranging from electronic health record and mobile disease self-management to artificial intelligence for the analysis of medical data and remote monitoring systems [6-8]. With this growth of technological possibilities for eHealth, the involvement of academics and stakeholders from the health, social, economic, legal, and data sciences and others has also increased [9]. This has led to a diverse set of experts being present in the field of eHealth research and development (R&D).

These varied stakeholders come from different disciplines; however, each field represents a relevant and necessary source of knowledge, making the fields dependent on each other [10,11]. For that reason, multidisciplinary collaboration is considered fundamental to the advancement of eHealth R&D [9,12]. Nevertheless, multidisciplinary collaboration does not occur effortlessly or without barriers as stakeholders may have contrasting levels of power and interest, which can lead to different objectives, priorities, and expectations [11,13,14]. Furthermore, due to the diversity in their background and expertise, stakeholders may not “speak the same language,” creating the potential for misunderstanding and conflict, which in turn may lead to suboptimal progress and outcomes [9,11]. These dynamics cause additional complexity in eHealth R&D and may impose higher management demands [11].

Thus, it is very important to engage and align stakeholders in constructive communication and cultivate relationships to facilitate this needed collaboration and ultimately attain the project objectives [15]. Stakeholders in eHealth R&D may benefit from a “shared design space” in which they reach a mutual understanding of each other’s worlds, including awareness of each other’s background, expertise, strengths, and perspectives [10]. However, much knowledge can be tacit, hidden in everyday practices and routines, or implicitly present as “common sense.” As a result, eHealth experts often end up working in parallel silos and may overlook opportunities for collaboration [9]. There is a need to create appropriate organizational room for communication and cooperation between different disciplines that facilitates the sharing of tacit knowledge as well [10].

Cocreation is an approach that is increasingly used in the field of eHealth to facilitate collaboration and bring forward tacit knowledge [16,17]. It is defined as “the collaborative generation of knowledge by academics working alongside stakeholders from other sectors” [18,19]. In eHealth R&D, cocreation is often used to involve end users, such as patients, to make participation in research more accessible and to collect end user input [20]. This is vital for eHealth innovations’ success as it makes services applicable to real-world settings [12,17]. However, other stakeholders should not be overlooked as an appropriate target for cocreation as it is an approach that may aid collaboration between disciplines and benefit multidisciplinary project management [14].

Previous studies have pointed out the current lack of practical guidelines that inform on the use of tools and methods, such as cocreation, for successful multidisciplinary collaboration [10,12,14]. Further research is necessary to identify and describe cocreation methods that can be used for this purpose. This paper, therefore, aims to add to the existing evidence base by providing insights into the application of cocreation, specifically in the early phases of research that focus on involving and aligning relevant stakeholders from different academic and professional backgrounds. This paper presents a case study of cocreative exercises conducted within the multidisciplinary CARRIER (Coronary Artery Disease: Risk Estimations and Interventions for Prevention and Early Detection) consortium and reports on the study’s practical experience and its implications. This may further improve the understanding of how cocreation can be used for multidisciplinary collaboration and encourage the uptake of cocreation for a wider audience than only end users.

## Methods

### Setting

The CARRIER consortium is a Dutch initiative in the South Limburg region that aims to reduce the burden of atherosclerotic cardiovascular disease (ASCVD) with the help of a personalized eHealth intervention. The consortium consists of experts in health and medicine, big data science, software development, and, lastly, ethical and legal experts in the medical domain. The objective of the project is to develop a big data-driven intervention to detect high-risk individuals, prevent cardiac events through health behavior changes, and ultimately reduce morbidity and mortality from ASCVD [21]. The content and delivery mode of the personalized eHealth intervention are to be developed by the consortium through cocreative design with end users and other stakeholders.

### Procedure

**Figure 1:**
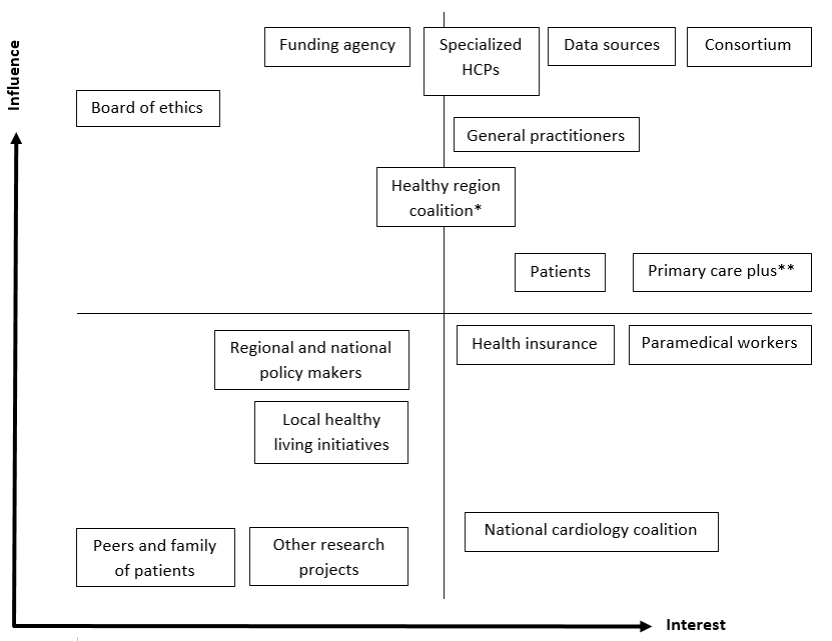
Power interest matrix for CARRIER. * Regional collaboration among health care, health insurance, knowledge institutes, and policy makers to create a healthy community. **Care organization between primary and hospital care. HCP: health care provider.

For this case study, the following 3 exercises were undertaken: a stakeholder mapping exercise, the development of a user scenario, and a group discussion based on the Six Thinking Hats of de Bono [22]. These exercises helped to determine with whom (stakeholder mapping), what (user scenario), and how (six hats method) the discussion should be undertaken. First, the health and medicine experts of the consortium conducted the stakeholder mapping exercise in preparation to facilitate the selection of relevant stakeholders for the Six Thinking Hats of de Bono discussion. No maximum number of participants was set beforehand. During this process, the team realized that, in this early phase of research and development, cocreation between colleagues was essential before reaching out to additional stakeholders, such as end users. Hence, no external stakeholders were asked to participate in the group discussion. Two web-based sessions were organized. In the first session, all possible stakeholders related to the CARRIER project were listed individually, compared, and grouped into 1 list. In the second session, the influence and interest of the stakeholders from the aforementioned list were discussed, and a power–interest matrix was produced (Figure 1). This matrix consisted of four categories such as (1) high influence, low interest; (2) high influence, high interest; (3) low influence, low interest; and (4) low influence, high interest. Each category represented a management strategy: (1) keep satisfied, (2) manage closely, (3) monitor, and (4) keep informed [18].

**Figure 2:**
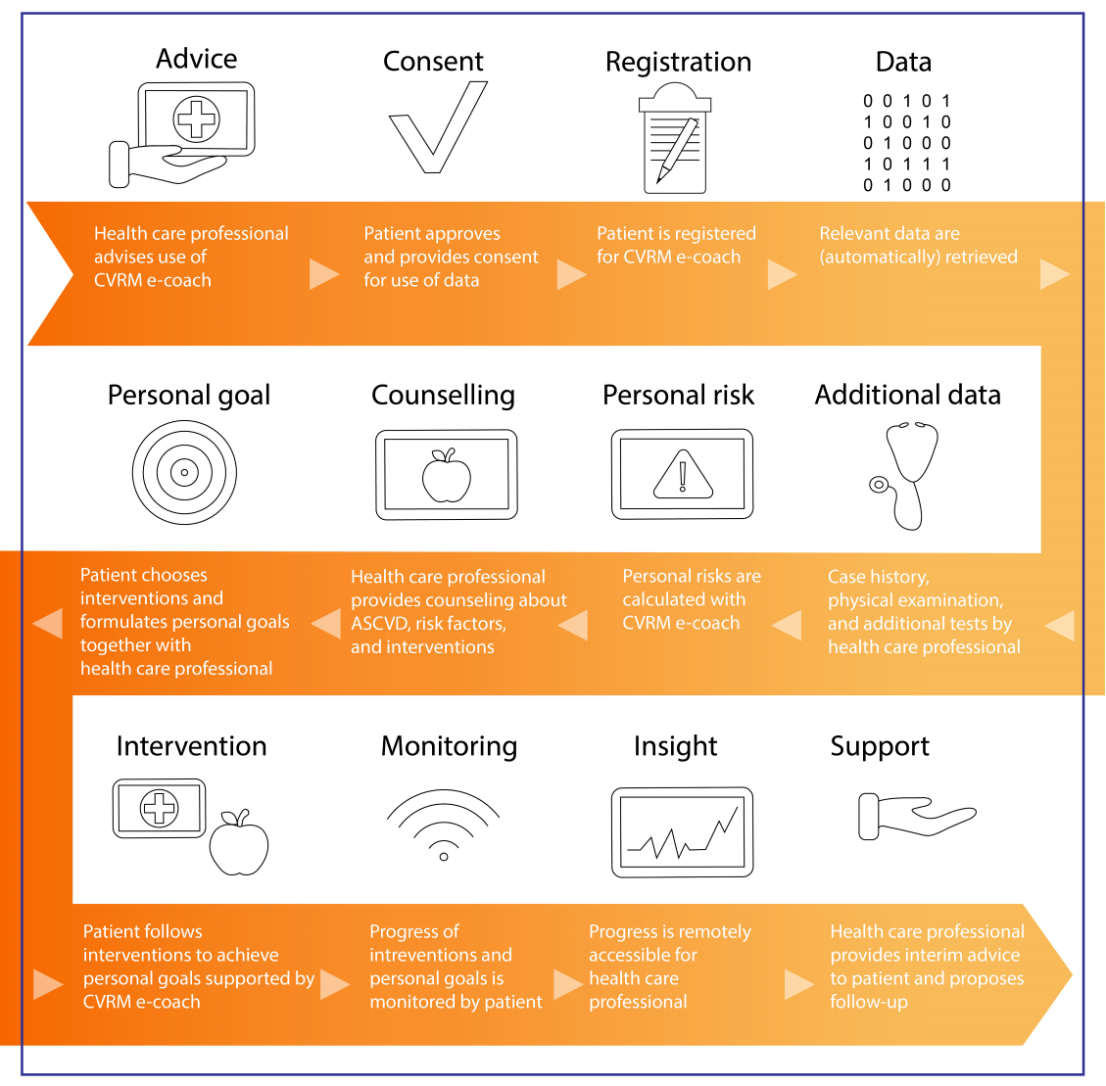
The dream user scenario. ASCVD: atherosclerotic cardiovascular disease; CVRM: cardiovascular risk management.

Subsequently, a user scenario was created by the authors to prompt conversation during the group discussion. This visual representation depicted the envisioned eHealth intervention in its ideal state and was therefore named the “dream” user scenario (Figure 2).

Lastly, the main exercise of this case study was a group discussion using the Six Thinking Hats of de Bono [22], which is a creative and solution-oriented method for brainstorming. The different thinking hats represent different viewpoints or so-called thinking directions and are used to facilitate lateral thinking. This method was chosen to engage and align the different stakeholders because it allows participants to share their experiences and expertise while also listening to and learning from each other. The 6 hats each have a color that corresponds to a particular thinking direction—informative thinking (white), intuitive thinking (red), positive thinking (yellow), critical thinking (black), creative thinking (green), and organizational thinking (blue). Informative thinking is meant to ensure objectivity, to collect existing knowledge or facts on the topic, and to determine what remains unknown. Intuitive thinking allows one to express thoughts based on emotions and intuition without the need for justification or judgment. Positive thinking comes from a place of optimism, aiming to explore opportunities or identify strengths and potential added value. Critical thinking, conversely, requires caution and careful consideration of the risks and barriers. The purpose of creative thinking is to be innovative and produce new ideas. Lastly, organizational thinking requires higher-level thinking, looking at the topic from a distance, and creating an overview and plan for the future. The 6 hats provide a framework for critical thinking that can be tailored to various contexts and audiences, ensuring its applicability in a wide range of scenarios. The flexibility of the methodology allows for multiple approaches. For example, hats can be assigned to specific participants, used collectively by all participants simultaneously, or interchanged among participants throughout the discussion. In this case, all the hats were used in the aforementioned order by all the participants at once, preventing confrontational discussion and making complex topics easier to discuss.

### Data Collection

For the discussion, 1 presenter (EL) and 1 discussion moderator (MS) were selected. The meeting started with a short introduction by each participant, followed by an explanation of the Six Thinking Hats of de Bono discussion structure. Then, the dream user scenario was presented and discussed from each of the 6 viewpoints. The meeting was organized digitally via videoconferencing and was scheduled to last 4.5 hours. All participants provided consent for the recording and processing of the full discussion. In addition, field notes were taken by both the presenter and the discussion moderator during the meeting to create a detailed summary of the discussion content, containing key comments from each participant per viewpoint. The summary was shared with the participants for member checking shortly after the discussion was conducted.

### Data Analysis

The analysis of the discussion content was carried out following a thematic approach, which is a method for identifying and describing patterns or reoccurring themes and consists of 6 steps [23]. The first step of data analysis involved becoming familiar with the collected data through transcription and reading. The recording was transcribed verbatim with the use of F4 transcription software. During the second step, the initial codes were generated independently by one of the authors (EL). In the third step, codes with similar content were clustered into an overarching theme per viewpoint. Next, in the fourth step, themes were compared and discussed between coauthors. The fifth step involved defining and specifying the themes to formulate suitable names. In the last step, the report was produced by selecting meaningful and representative quotes to function as examples. Qualitative analysis of the transcript was carried out with the use of ATLAS.ti software (ATLAS.ti Scientific Software Development GmbH).

### Ethical Considerations

Ethical approval for this research project was waived by the Medical Ethical Testing Committee (METC) of Maastricht University and Maastricht University Medical Centre as this study did not meet the criteria for the Medical Research Involving Human Subjects Act (METC 2019-4792).

## Results

### Participants

Eight stakeholders, consisting of 4 health and medicine experts, of whom 2 were cardiologists and 2 were health service researchers, 2 software developers, 1 data scientist, and 1 innovation manager, were invited to participate in the Six Thinking Hats of de Bono discussion. The participants’ characteristics are presented in Table 1.

The outcome of the discussion is described below by viewpoint, and a summary of the themes per viewpoint is presented in Table 2. The first 2 viewpoints (informative and intuitive thinking) served a preparatory purpose, enabling individuals to familiarize themselves with the details of the topic and the other participants. Then, the body of the discussion consisted of the middle 2 viewpoints (positive and critical thinking). These viewpoints resulted in an in-depth discussion and were therefore the most time-consuming viewpoints. Lastly, the 2 remaining viewpoints (creative and organizational thinking) were action oriented, building upon the outcomes of the previous viewpoints. Here, the focus was on solutions and planning to safeguard future progress.

**Table 1:** Characteristics of the participants.

Characteristics		Values
**Gender, n (%)**		
	Female	4 (50)
	Male	4 (50)
Age (years), mean (SD)		40.4 (8.7)
**Field of expertise, n (%)**		
	Data science	1 (12.5)
	Software development	2 (25)
	Health and medicine	4 (50)
	Innovation management	1 (12.5)
Years of work experience, mean (SD)		15.6 (8.0)

**Table 2:** Themes per viewpoint.

	Informative	Intuitive	Positive	Critical	Creative	Organizational
Intervention content	✓			✓	✓	✓
Functionalities	✓	✓		✓	✓	✓
Implementation in practice	✓	✓	✓	✓		✓
Legal requirements		✓		✓		✓
Use of big data			✓	✓	✓	✓
Stakeholder management			✓	✓		
Consortium impact			✓			
Time and budget constraints				✓		
Alternative design					✓	

### Informative Thinking (White)

Regarding the *intervention content*, the proposed domains for behavioral change modules in the dream user scenario (ie, medication adherence, smoking cessation, physical activity, healthy diet, and coping with stress) were deemed sufficient. The health and medicine stakeholders inquired of the software development stakeholders whether the actual content of these modules was ready to use, whether it was still to be developed, or whether links should be made with existing external initiatives. Furthermore, the software development stakeholders wondered about potential strategies that the health and medicine stakeholders may have that could ensure patient engagement and obtain long-term lifestyle improvements. In the case of *functionalities*, the data science stakeholders were asked questions about the prediction model mechanism, how one could interact with the model, and what the impact of missing variables would be. Clarification was also requested by the health and medicine stakeholders regarding the possibility of combining multiple behavior change goals (eg, diet and physical activity) and incorporating wearables for monitoring purposes (eg, heart rate). With respect to *implementation in practice*, the software development and innovation management stakeholders wondered how many different HCPs would be involved in the intervention, which HCP would be the most suitable to take the lead, and to whom the online environment with patient data would be accessible.

Another challenge is [..], how do we motivate patients to change behavior, how do we monitor it and how do we keep them on track? That’s part of our expertise of course, but I think we need to do more than what we have done in the past.

Software development stakeholder

### Intuitive Thinking (Red)

Stakeholders unanimously agreed on the project being ambitious, innovative, and relevant, though concerns were expressed about realizing the dream user scenario. As for *functionalities*, the software development and health and medicine stakeholders found health education, goal setting, monitoring, and feedback to be essential components. Furthermore, health and medicine stakeholders wished to have the intervention integrated into a universal web-based platform that is both compatible with other systems as well as adaptable when changes are needed. Concerning *implementation in practice*, a blended care format in which patients receive both in-person and digital health services was favored by all the stakeholders as it may help to facilitate shared decision-making, to reach all patients regardless of their digital literacy, and to reduce dropout. Lastly, the software development and management stakeholders stressed the importance of *legal requirements* and the need to take protocols and legislation, such as CE certification and privacy issues, into consideration.

I do think it’s innovative, there is a big challenge and also a big improvement for the patients at target, but it’s also very ambitious because we have different stakeholders to manage and barriers we need to survive.

Management stakeholder

### Positive Thinking (Yellow)

For *implementation in practice*, stakeholders again mentioned the importance of blended care as it creates the opportunity to supervise patients and support the continuity of eHealth use. The health and medicine stakeholders mentioned that the intervention should not compete with or disturb the current in-person or digital practices but rather complement them. All the stakeholders recognized that the development process provides room and flexibility to incorporate valuable input from all the partners involved. Therefore, the consortium wanted to seek opportunities for collaboration to align the development with practice. Hence, the availability of multidisciplinary expertise within the consortium and its network was greatly appreciated (*stakeholder management*). The health and medicine stakeholders also discussed the vast amount of useful, yet underused, data (*use of big data*) that is present in hospitals and other institutions, creating substantial opportunities for medical and prevention purposes, such as individual risk calculations. An effective tool, on the one hand for changing health behavior and reducing ASCVD risk and on the other for transferring care from the hospital to the home setting, may be exemplary for other patient groups. Accordingly, the participants considered the project as a stepping stone for future innovations (*consortium impact*), even without fully realizing the dream scenario.

The opportunities are great because (…) my patient files are doing nothing for me, I just have to look up the information and I have to construct my own risk model each and every time. So if that could be integrated, it could be fast and ready and at my fingertips.

Health and medicine stakeholder

### Critical Thinking (Black)

Intervention content was discussed again by the software development stakeholders as challenges were identified for personal risk communication; more specifically, these were how to communicate in an understandable and motivating manner to induce behavior change and, for the modules, particularly how to transform content with personalized and motivational features to ensure actual behavior change. With regard to *functionalities*, automatic data collection for calculating personal risks was requested by the health and medicine stakeholders to create an easy-to-use intervention that is less susceptible to errors. As (local) institutions have to share big data (*use of big data*) while complying with *legal and ethical regulations*, automatic data collection might only be partially possible. This may lead to a less user-friendly tool. *Time and budget constraints* also formed an important part of the discussion as these had an impact on all the consortium’s activities. According to both the software development and the health and medicine stakeholders, more financial resources are needed for the development of new content. For the *implementation in practice*, the health and medicine stakeholders stressed the essence of reimbursement. Without a financial structure, sustainable implementation will become challenging. In terms of *stakeholder management*, although the diversity in expertise was previously seen as positive, it was also pointed out that each stakeholder has their own objectives; hence, creating value for each party could become difficult. Due to large interdependencies between working groups, a delay in activities by 1 stakeholder (eg, building and training the prediction model) directly influences the subsequent activities of another stakeholder (eg, usability, feasibility, and impact evaluation), thereby creating barriers to project planning.

I have concerns that the risk communication won’t work and that patients will just see a number or eh ... you know whatever the app says and that they will just ignore it and just keep as they are doing.

Data science stakeholder

### Creative Viewpoint (Green)

Regarding the *intervention content*, the personalization of the modules was the main focus in the project. To this end, the health and medicine stakeholders will conduct research on personalization strategies as well as understand the preferences and needs of end users regarding personalization. The findings will serve as a guide for the development process of the content. As discussed earlier, a prominent challenge for the consortium was the combination of time and budget constraints and the need for new personalized and motivating modules. Hence, an *alternative design* was discussed, in which patients would be educated on diagnosis and related risk factors, including personalized risk communication, as well as being given an overview of potentially relevant behavior change interventions to choose from, while receiving monitoring and feedback *functionalities*. This intervention referral or decision aid set up would safeguard the project’s aim. Lastly, as automatic data collection (*use of big data*) might only be partially possible, a risk assessment questionnaire—to be filled in manually by patients or HCPs—was proposed by the data science stakeholder.

Prevent reinventing the wheel! We have care providers that do excellent smoking cessation sessions and those that provide great dietary interventions and or make you exercise more. Ideally, the eHealth platform should be able to connect those health care providers to the specific patient who may benefit most from that intervention.

Health and medicine stakeholder

### Organizational Viewpoint (Blue)

The majority of future steps consisted of new appointments for an in-depth discussion of creative solutions, challenges, or opportunities. For the *intervention content*, the software development stakeholders need to clarify the extent to which the required content is already available and what still needs to be added. At the same time, the health and medicine stakeholders will explore possibilities to make use of content within existing (eHealth) interventions. Furthermore, the intervention *functionalities* require further discussion to specify the features that are needed and wanted according to the health and medicine stakeholders as well as feasible to incorporate into the future eHealth intervention according to the software development stakeholders. This also includes considerations of the patient pathway, meaning how the intervention will be used by the patient and HCP end users when *implemented in practice*. Lastly, as the *use of big data* has legal and ethical implications, the data science stakeholders agreed on a joint follow-up meeting with both legal experts and innovation managers of local hospitals to have an in-depth discussion on the *legal requirements* and system integration. Furthermore, the data science stakeholder will provide clarity on the possibility of automatic, semiautomatic, or manual data entry for risk calculation, which will also inform the intervention design in the future. “I tried to make main themes that I think we have to work on and maybe we can make new arrangements for that” [Health and medicine stakeholder].

## Discussion

### Principal Findings

The aim of this paper was to contribute to the existing evidence base by contextualizing cocreation for involving and aligning relevant stakeholders in the early phases of a multidisciplinary research project. This paper presented a case study on the Six Thinking Hats of de Bono discussion method and reported on the outcome. The colored “thinking hats” served as a simple metaphor and invited participants to “change their hats” to view a topic from multiple viewpoints instead of holding onto 1 perspective. Nine themes such as intervention content, functionalities, implementation in practice, legal requirements, use of big data, stakeholder management, consortium impact, time and budget constraints, and an alternative design were identified. All the themes were discussed from the critical viewpoint, that is, risks and barriers, except the consortium impact and the alternative design. Previous research has found similar challenges; hence, these themes may represent common barriers in eHealth development [24-27].

To overcome the lack of relevant intervention content, more specifically the lifestyle modules, it was proposed to use existing lifestyle interventions. Basically, this concept can be compared with a patient decision aid in which patients and HCPs are guided to the best prevention option depending on contextual factors. This approach will provide guidance for selecting the most appropriate lifestyle interventions from the current options that are available and suitable. Many different digital tools are already in place for a variety of health-related purposes. However, potentially unhealthy factors of this digital transformation are becoming apparent, such as digital overload and digitization-related stress, which negatively affect well-being [28]. This mainly applies to the work environment and thus HCPs, although it may relate to patients as well. In the health care setting, it has been argued that it is not so much the digital overload but rather “filter failure” (ie, the inability to navigate the abundance of information available in digital spaces) that causes problems [29]. A so-called lifestyle decision aid would prevent this surplus from expanding and help to navigate the existing digital tools and information instead, benefitting both providers and patients.

Furthermore, reduced use over the course of time or complete dropout are common phenomena for eHealth apps [30]. Hence, blended care was preferred for the delivery of the current eHealth intervention as a strategy to safeguard patient engagement. Research has indeed shown that the involvement of a supervising HCP increases adherence to an eHealth intervention when compared with independent use [31]. Furthermore, blended care was seen as important to enable integration into regular in-person services. However, the implementation of digital health services has been recognized as a complex process that relies on several prerequisites. These include enabling the active participation of end users during the development process, minimizing disruptions to existing workflows, and ensuring that the solution effectively resolves a concrete issue or provides value in general in situations in which there is no explicit problem to be solved [32,33].

Lastly, budget and time constraints may appear to be a less prominent topic; however, they constituted an important part of the discussion due to their implications for all aspects of the project. Robust research requires financial resources from grants or other sources and, above all, time to be conducted rigorously. Even though academia is appreciated for knowledge and innovation, the academic environment may appear slow to take action and thus be less attractive for collaboration than industry partners [34]. However, the ongoing trends in health care, such as the rise of chronic diseases, the need for a sustainable workforce, and financial challenges, are presenting us with highly complex and interconnected issues, which are also known as wicked problems. Addressing these wicked problems requires collaborative efforts and innovative strategies that consider diverse perspectives and engage various fields of expertise [35]. Health research will become ever more interdisciplinary and dependent on cooperation with other nonmedical or nonscientific disciplines, demanding a new approach to working that may feel unfamiliar*.* This makes it crucial to understand why and how some multidisciplinary groups fail, struggle, or succeed in delivering tangible outcomes. Translating these experiences into general lessons will provide insights into contextual and human factors, such as relevant skills and organizational characteristics. These will help to build better collaborations in the future and to achieve better outcomes.

### Lessons Learned

This case study described the structure and specific purpose that the Six Thinking Hats of de Bono can provide when applied to a group discussion. With the help of the stakeholder matrix, the right people were involved at the right time and the dream user scenario made tacit knowledge explicit and created opportunities for shared decision-making. Some reflections can be made on safeguarding the process and realizing positive results. The recommendations for conducting a Six Thinking Hats of de Bono group discussion with multidisciplinary stakeholders are summarized in Textbox 1. First, a group discussion requires active participation. The participants were briefly informed of the discussion approach and content; however, no details were shared prior to the meeting. This created the possibility of discussing first impressions and prevented the participants from preparing socially desirable statements. Common issues with interactive group work, such as fear of negative evaluation, relying on others to contribute, and matching the least productive performance, need to be managed [36-38]. Therefore, appointing a discussion moderator ensures that all the participants engage in the conversation and follow the determined structure of thinking hats. A moderator may also help to create a safe space for honest and open communication.

**Recommendations for a Six Hats discussion with multidisciplinary stakeholders.**

1. Specify the purpose or aim of the discussion.

2. Invite relevant stakeholders from different disciplines.

3. Determine the topic of discussion.

4. Prepare preferably visual content to introduce the topic of discussion.

5. Establish what, if any, structure the discussion will follow.

6. Determine an acceptable timeline for the discussion.

7. Appoint a discussion moderator and, optionally, a note-taker.

8. Be mindful about creating a safe and collaborative space.

Next, preparing discussion content in advance is also recommended for facilitating active participation so that the main theme of the conversation is clear. Moreover, such complementary content can be beneficial by serving as a starting point, icebreaker, or probe for conversation. The dream user scenario, for example, provided a comprehensible visual representation of the project. This directed attention to the complexities that needed consideration, generating relevant topics for conversation and overcoming the language gap [9,24]. The use of visualizations can improve the performance of cognitive, communicative, and collaborative tasks [39]. A previous study has indeed found that visualizations are significantly better than text for attracting attention, achieving agreement, and ensuring information retention [40]. Depending on the objective, other visualization tools besides user scenarios, such as explorative prototypes [41], health systems mapping [42], and mind mapping can be used [43].

Finally, all 6 hats were used in a predetermined sequence to consider the topic from all perspectives and provide structure to the conversation. Each participant was offered the opportunity to speak for each viewpoint, leading to a better mutual understanding and realistic expectations for the future of the project. However, this is not a requirement. Depending on the discussion aim, the 6 hats may also be used freely as needed spontaneously and do not have to be used all at once. In this case study, all the thinking directions were conducted consecutively, which showed itself to be a time-consuming exercise, and one may consider organizing several sessions instead. Nevertheless, the experiments by Göçmen and Coşkun [44] demonstrated that intentional time limitations during a Six Thinking Hats of de Bono discussion lead to more creative and unique ideas. Therefore, setting time limitations may actually be helpful for creative thinking specifically. It is not only an easy-to-use method but also adaptable for different targets and target audiences. Hence, other papers have recommended this method for a variety of purposes, such as collaborative care [45], relationship counseling [46], work meetings [47], and education [48].

### Limitations and Strengths

Many publications have described the eHealth development process, that is, reporting on their iterations toward a final product or service; yet, only a few have provided in-depth reflections on the development process itself, such as the experienced barriers or facilitators [15]. Nevertheless, there is a need for such information to improve multidisciplinary working in eHealth and other fields [10,11,14]. This paper provided a detailed explanation of such a “tool,” its application, and its outcomes based on a real-world case from a complex multidisciplinary eHealth consortium. Therefore, the application and process of cocreation and the subsequent practical lessons can be considered a strength. A limitation arising from this descriptive approach is that no qualitative or quantitative data were collected on the participants’ self-reported experiences. Although data on satisfaction with the method used or perceived effectiveness could have provided useful insights, the sole aim of this paper was to present the application of a specific cocreation method to project management and not to evaluate it. Lastly, not all stakeholder groups were invited to participate in this exercise, which could be considered a limitation. However, at this point in time, the aim was to engage and align the stakeholders on the possibilities of the project and adopt a project management focus. This was a preparatory exercise conducted early in the research to prevent confusion and promote efficiency in future interactions with other stakeholders such as patients. In addition, sufficient opportunity for stakeholder participation and input remains, as well as project flexibility to incorporate new knowledge.

### Conclusions

This paper has demonstrated how cocreation can be applied to stakeholder involvement and alignment in practice. More specifically, the case has shown how the Six Thinking Hats of de Bono method can be a straightforward, low cost, and adaptable tool to overcome common barriers in multidisciplinary research environments and facilitate collaboration. It is recommended to create a stakeholder overview and the discussion content in advance and appoint a moderator to facilitate active participation as well as a safe environment. The discussion, in combination with visual communication, helped to make tacit knowledge explicit, identify points for improvement, and remain solution oriented. More evidence on contextual and human factors, such as relevant skills and organizational characteristics, will help to build better collaborations, and thus outcomes, in the future of multidisciplinary research.

## Abbreviations

ASCVD: atherosclerotic cardiovascular disease
CARRIER: Coronary Artery Disease: Risk estimations and Interventions for prevention and Early Detection
HCP: health care provider
METC: Medical Ethical Testing Committee
R&D: research and development
